# Quality of antenatal care service provision in health facilities across sub–Saharan Africa: Evidence from nationally representative health facility assessments

**DOI:** 10.7189/jogh.07.021101

**Published:** 2017-12

**Authors:** Mufaro Kanyangarara, Melinda K Munos, Neff Walker

**Affiliations:** Institute for International Programs, Johns Hopkins Bloomberg School of Public Health, Baltimore, Maryland, USA

## Abstract

**Background:**

Utilization of antenatal care (ANC) services has increased over the past two decades. Continued gains in maternal and newborn health will require an understanding of both access and quality of ANC services. We linked health facility and household survey data to examine the quality of service provision for five ANC interventions across health facilities in sub–Saharan Africa.

**Methods:**

Using data from 20 nationally representative health facility assessments – the Service Provision Assessment (SPA) and the Service Availability and Readiness Assessment (SARA), we estimated facility level readiness to deliver five ANC interventions: tetanus toxoid vaccine for pregnant women, intermittent preventive treatment for malaria in pregnancy (IPTp), syphilis detection and treatment in pregnancy, iron supplementation and hypertensive disease case management. Facility level indicators were stratified by health facility type, managing authority and location, then linked to estimates of ANC utilization in that stratum from the corresponding Demographic and Health Surveys (DHS) to generate population level estimates of the ‘likelihood of appropriate care’. Finally, the association between estimates of the ‘likelihood of appropriate care’ from the linking approach and estimates of coverage levels from the DHS were assessed.

**Findings:**

A total of 10 534 health facilities were surveyed in the 20 health facility assessments, of which 8742 reported offering ANC services and were included in the analysis. Health facility readiness to deliver IPTp, iron supplementation, and tetanus toxoid vaccination was higher (median: 84.1%, 84.9% and 82.8% respectively) than readiness to deliver hypertensive disease case management and syphilis detection and treatment (median: 23.0% and 19.9% respectively). Coverage of at least 4 ANC visits ranged from 24.8% to 75.8%. Estimates of the likelihood of appropriate care derived from linking health facility and household survey data showed marked gaps for all interventions, particularly hypertensive disease case management and syphilis detection and treatment. There was fairly good concordance between our estimates of high likelihood of appropriate care and DHS estimates of coverage for iron supplementation, IPTp, and tetanus toxoid vaccination.

**Conclusion:**

Linking household surveys to health facility assessments revealed marked gaps in population–level coverage of quality ANC interventions and underscored the need for a double–pronged approach to increase ANC utilization and improve the quality of ANC services.

High quality antenatal care (ANC) can substantially reduce maternal and newborn mortality [1,2]. High population–level coverage of ANC is necessary to deliver proven interventions to improve maternal, newborn and child health (MNCH). Globally, progress to achieve universal ANC coverage is monitored by tracking two indicators: 1) the proportion of women of reproductive age who report at least one ANC visit with a skilled health provider during the most recent live birth (ANC 1+); and 2) the proportion of women of reproductive age who report at least four ANC visits with any provider during the most recent live birth (ANC 4+) [3,4]. These two indicators quantify the number of contacts pregnant women in low– and middle–income countries have with the existing health infrastructure. Contact with the health system does not guarantee the receipt of all or any of the necessary ANC interventions. However, in theory, the more points of contact that a woman has with a well–functioning and effective health system during pregnancy, the greater the opportunity and higher the likelihood that potential pregnancy complications will be prevented, detected, and treated in a timely manner.

Uptake of ANC services has steadily increased over the past two decades according to trends from population–based surveys. Countdown to 2015, a multi–stakeholder collaboration tracking country–level coverage for MNCH, reported that the majority of women (90%) have at least one ANC visit, while only 57% have the recommended four or more ANC visits [5]. As ANC utilization increases, simply reporting on ANC1+ and ANC4+ is insufficient to help countries achieve their maternal health objectives. A country may have high levels of ANC4+ but few women receiving the recommended ANC interventions if the quality of ANC services is poor. Conversely, better service provision is unlikely to improve health outcomes if uptake is low. Service provision and service utilization have often been considered in isolation of each other, providing a fragmented picture of the effectiveness of interventions within a health system. Continued gains in maternal and newborn health will require countries to measure and link both ANC contacts and content in order to inform improvements in access to and quality of ANC services [4,6,7].

Large scale cross–sectional household surveys such as the Demographic and Health Surveys (DHS) and the Multiple Indicator Cluster Surveys (MICS) provide measures of ANC1+ and ANC4+, as well as limited information about physical examinations conducted during ANC [8,9]. These surveys ask women about whether their height, weight and blood pressure were ever measured during ANC, whether urine and blood samples were collected, whether tetanus toxoid vaccination and intermittent preventive treatment of malaria in pregnancy (IPTp) were given, and whether women received counselling on danger signs for pregnancy complications. Multi–country assessments conducted to evaluate the content of ANC visits have found that estimated coverage of these components of ANC was lower than expected based on ANC1+ and ANC4+ coverage due to poor quality of care [5,6,10,11]. An analysis of DHS data from 41 countries showed that the coverage of eight recommended ANC components ranged widely and averaged only 60% among women who reported attending at least four ANC visits [6]. Population–based household surveys may adequately monitor the number of ANC visits and the receipt of simple procedures such as the collection of urine and blood specimens for screening. However, it is unclear whether respondents can correctly recall more complex procedures, and details about examinations or laboratory investigations conducted during one or more ANC visits. The scope and type of ANC interventions that can be reliably tracked using household surveys may be considerably limited. Additionally, the survey reference period often spans up to five years, which calls into question issues related to measurement error or recall bias. Combining service utilization data from household surveys with service provision data from health facility assessments may be a useful strategy to improve coverage measurement for MNCH [12–14].

Health facility assessments are sample surveys or censuses of health facilities designed to assess and monitor the status of the health system. They typically assess service availability (the physical presence or reach of health facilities) and service readiness (the capacity to deliver the services offered) [15]. The availability of trained and supervised staff, guidelines, equipment, diagnostics, medicines and commodities, determined through both self–report and direct observation, form the basis of monitoring and evaluating service–specific readiness [16]. The underlying assumption is that the availability of these elements is a prerequisite for the delivery of high–quality care, and that examining readiness is critical for understanding service quality. Although several previous studies have explored the readiness of health facilities to provide services for ANC [17–19], child health [20], tuberculosis treatment [21], family planning [22,23] and essential medicines [24], only a few have linked estimates of readiness to population–based care–seeking estimates from household surveys to estimate coverage of MNCH interventions [25–27].

The objective of this study was to characterize the likelihood of appropriate care for different ANC interventions across sub–Saharan Africa by linking data from nationally representative health facility assessments to nationally representative household surveys. This study also highlighted the feasibility and methodological challenges of linking health facility assessments to household surveys.

## METHODS

### Data sources

This study relied on three primary data sources: the Service Provision Assessments (SPA), the Service Availability and Readiness Assessments (SARA) and the Demographic and Health Surveys (DHS). The SPA survey, developed by ICF International, is a cross–sectional health facility assessment that aims to systematically review the status of health systems by examining infrastructure, equipment, and supplies considered necessary to provide quality health services [28]. The sampling frame for the survey is the national master facility list, a complete listing of all public and often non–public health facilities in the formal sector ranging from primary to tertiary level of care. Tertiary level hospitals are often oversampled. Typically, SPA surveys adopt a sampling design that allows the calculation of nationally representative indicators by region, health facility type, and managing authority. In countries with a limited number of health facilities or where the budget permits, a full census approach has been used. The core data collection tool, a facility inventory questionnaire, is used for the direct verification of the availability of drugs, commodities, equipment and amenities. Details about the survey design and survey procedures are described in the final survey reports issued by the respective countries. Full survey data sets are available in the public domain [29].

Similar to the SPA, the SARA is a nationally representative assessment of health facilities on the availability and readiness to provide essential health services. The SARA tool was developed in 2008 by a collaboration of the World Health Organization (WHO) and the United States Agency for International Development (USAID) [16]. Building on experiences and best practices from other health facility assessments including the SPA, the SARA was designed to serve as a rapid health facility assessment tool to track quality of health services annually and inform national health planning. The SARA framework is based on a common set of indicators and summary measures formulated to detect changes and monitor progress in health systems strengthening, while enabling comparisons across facilities. Often, random stratified sampling of health facilities by health facility type and managing authority is used to select a nationally representative sample from a national master facility list. Detailed descriptions of the survey process, methodology and data collection instruments are available online [30].

The DHS is a nationally representative household cluster survey used to collect data on sociodemographic characteristics, family planning, fertility, and maternal and child health. The women’s module, administered to women aged 15–49 years, collects details on whether ANC was sought and the source of ANC (facility type) for the most recent pregnancy that led to a live birth within the five years preceding the survey. Women also report on the basic components of ANC received, including weight and height measurement, blood pressure measurement, blood and urine specimen collection, iron supplementation, malaria prophylaxis in malaria endemic countries, tetanus toxoid vaccination, and counselling about potential pregnancy complications. To limit recall bias, our analysis was restricted to the sample of women aged 15–49 years who reported a live birth in the three year period preceding the index survey. Similar to the DHS, MICS collect information detailing ANC services received. However, MICS data sets were excluded from this analysis because they do not collect data on the type of health facility where women sought ANC, which is necessary for the linkage.

Our analysis included countries in sub–Sahara Africa with at least one SPA or SARA survey conducted in or after 2000, and with a corresponding DHS conducted within 2 years (prior to or after the SPA/SARA). Multiple health facility surveys were available and included for several countries (Democratic Republic of Congo (DRC), Kenya, Senegal, Sierra Leone, Tanzania and Uganda).

### Analysis

**Figure 1:**
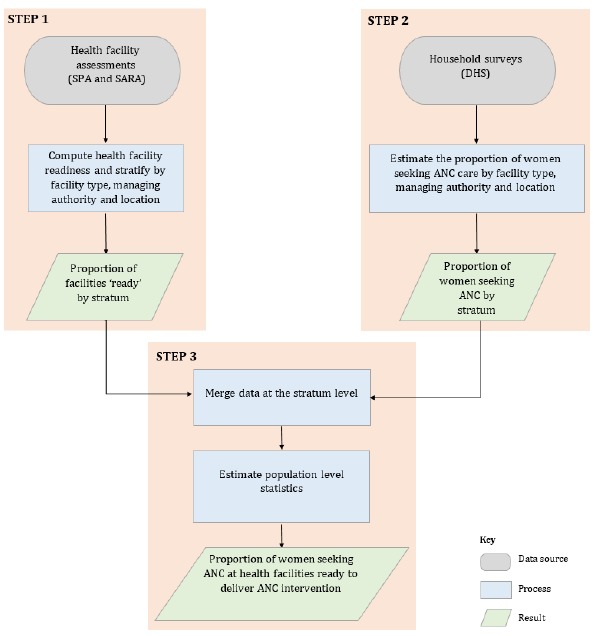
Three–step process to link health facility data (SPA and SARA) to household survey data (DHS).

The analysis used a three step process to link health facility data from SPAs and SARAs to DHS household survey data (**Figure 1**). In Step 1, five key ANC interventions were chosen based on the availability of information on the particular intervention components which could be standardized across both the SPA and SARA. The ANC interventions included: (i) tetanus toxoid vaccine for pregnant women; (ii) intermittent preventive treatment of malaria in pregnancy (IPTp); (iii) syphilis detection and treatment in pregnancy; (iv) hypertensive disease case management, including management of pre–eclampsia with magnesium sulphate; and (v) iron supplementation. We defined the components of each intervention and made adjustments to align definitions across the SPAs and SARAs (**Table 1**). While coverage of prevention of mother–to–child transmission of HIV (PMTCT) services is important in the sub–Saharan Africa context, the intervention was excluded as most of the health facility surveys only assessed whether ANC facilities offered PMTCT services, and no further verification of the availability of components of PMTCT was conducted.

**Table 1:** Definition of indicators of health facility readiness to deliver key antenatal care (ANC) interventions

Domain	Indicator	Definition of indicators
Staff and guidelines	Staff trained in ANC	▪ At least one staff member trained in at least one aspect of ANC
Guidelines on ANC	▪ Observed or reported the availability of ANC guidelines
**Equipment, diagnostics, medicines and commodities**	Tetanus toxoid vaccine for pregnant women	▪ Observed at least one valid unexpired unit of tetanus toxoid vaccine
	Intermittent preventive treatment of malaria in pregnancy	▪ Observed at least one valid unexpired unit of sulphadoxine/pyrimethamine
	Syphilis detection and treatment in pregnancy	▪ Observed at least one valid syphilis test† ▪ Observed at least one valid unexpired unit of medicine to treat syphilis‡
	Hypertensive disease case management	▪ Observed at least one valid dipstick for urine protein OR acetic acid and flame for heating ▪ Observed at least one functioning blood pressure apparatus§ ▪ Observed at least one valid unexpired unit of magnesium sulphate
	Iron supplementation	▪ Observed at least one valid unexpired unit of iron or iron and folic acid tablets

*Data on receipt of training of ANC services had varying recall periods ranging from 1–3 years preceding the survey.

†Syphilis rapid diagnostic test (RDT), Venereal Disease Research Laboratory (VDRL) test or polymerase chain reaction (PCR) or rapid plasma reagin (RPR).

‡ Injectable benzathine penicillin or procaine penicillin.

§Automatic or manual with stethoscope.

For each intervention, indicators of readiness to provide the intervention on the day of the health facility assessment were calculated for each health facility that reported offering ANC services. In facilities with multiple pharmacies or medical storage rooms, a medicine was considered available if at least one unit with a valid expiration date was observed in any location. Similarly, diagnostic services and equipment were considered available if they were available and functioning in at least one location or service area. Missing values were treated as indicating the unavailability of the component of interest and accounted for <1% of the sample size for all interventions by country.

Facility–level indicators were stratified by health facility type (hospital/ health center/ health post, etc.), managing authority (public/non–public) and location (urban/rural) to obtain stratum–specific proportions of ANC facilities ‘ready’ to provide each intervention. These stratification variables were selected to account for hypothesized differences in the service environment by health facility type and heterogeneity in health care seeking behaviors by location (urban/rural). Information on the location stratum was missing for SPA surveys conducted prior to 2009, in which case only health facility type and managing authority were used for stratification.

In Step 2, among women aged 15–49 years who reported attending at least four ANC visits (ANC4+) in the DHS survey, source of ANC was categorized as above. For women providing multiple responses about where they sought ANC, only the highest level facility type was included. In the final step, the proportion of women attending ANC4+ in each facility stratum was merged with summary statistics of readiness derived from the SPA/SARA, using facility stratum as the identifier. For each intervention, the expected proportion of women attending ANC at health facilities ‘ready’ to provide the intervention at the national level was estimated by summing, across strata, the proportion of ANC facilities ‘ready’ to provide the intervention in that stratum (from the SPA/SARA) multiplied by the proportion of women who reported attending at least four ANC visits in that stratum (derived from the corresponding DHS).

We categorized women attending at least four ANC visits into three groups based on the “likelihood of appropriate care”: (i) high likelihood of appropriate care if they attended a health facility type that in the SPA/SARA had the necessary equipment, diagnostics, medicines and commodities in stock, was equipped with ANC guidelines, and had at least one staff member who had been trained recently; (ii) moderate likelihood of appropriate care if they attended a health facility type that in the SPA/SARA had the necessary equipment, diagnostics, medicines and commodities in stock, but the health facility type lacked ANC guidelines or trained staff members (or both); and (iii) low likelihood of appropriate care if they attended a health facility type that in the SPA/SARA did not have the necessary equipment, diagnostics, medicines and commodities in stock, regardless of the availability of ANC guidelines or trained staff members. This classification encompasses all four domains that are required for service specific readiness within the SARA framework – availability of guidelines and trained staff, equipment, diagnostics, and medicines and commodities [16].

We conducted a sensitivity analysis to check the robustness of the overall results to misclassification of the source of ANC. Two hypothetical cases were considered. The upper bound corresponded to the hypothetical scenario in which all women attending at least four ANC visits were assumed to have sought ANC at health facilities in the stratum with the highest level of readiness for each intervention. Conversely, the lower bound was obtained by assuming all women attending at least four ANC visits sought care at health facilities in the stratum with the lowest level of readiness.

Finally, to assess the plausibility of our results, we compared our results to self–reported receipt of ANC interventions for those interventions where DHS surveys are used to estimate coverage based on maternal recall (tetanus toxoid vaccine, IPTp, and iron supplementation).

All analyses accounted for the complex survey design and adjusted for sampling weights. Analyses were conducted using STATA 14 (College Station, TX).

## RESULTS

**Figure 2:**
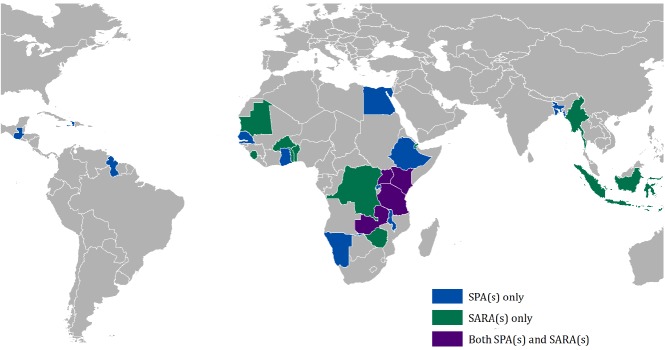
Geographic distribution of Service Provision Assessment (SPA) and Service Availability and Readiness Assessment (SARA) surveys.

A total of 44 health facility assessments were identified, with the majority (36/44, 82%) in sub–Saharan Africa (**Figure 2**). Of the 36 health facility assessments conducted in sub–Saharan Africa, 1 was excluded as it was conducted prior to 2000, 9 were excluded as there was no corresponding DHS within two years of the health facility assessment, and 6 were excluded as they did not contain information on ANC services. The final sample included 20 assessments that reported on a total of 10 534 health facilities, of which 8742 (82.9%) reported offering ANC services. The percentage of facilities offering ANC services ranged from 73.9% to 95.3% across the 20 health facility assessments (**Table 2**).

**Table 2:** Indicators of health facility readiness to deliver key antenatal care (ANC) interventions from the SPAs and SARAs*

Country	Year	Survey	Total number of health facilities	Facilities offering ANC services (%)	ANC facilities with staff trained in ANC(%)	ANC facilities with guidelines on ANC (%)	Percentage of ANC facilities with the equipment, diagnostics, medicines and commodities to deliver				
**TT**	**IPTp**	**Syph T&T**	**Hyp**	**Fe**
Benin	2013	SARA	189	83.6	49.9	70.8	68.8	78.5	34.9	34.9	96.9
Burkina Faso	2012	SARA	686	88.8	71.8	94.2	91.5	51.5	4.6	15.4	88.0
DRC	2013	SARA	317	91.8	62.3	75.7	28.0	97.0	11.3	3.6	62.4
DRC	2014	SARA	1555	73.9	45.3	47.3	26.3	63.3	34.3	6.9	59.7
Ghana	2002	SPA	428	87.1	53.8	55.7	77.4	37.4	3.1	65.2	81.9
Kenya	2004	SPA	440	79.3	68.9	33.2	83.6	96.6	41.4	4.2	86.2
Kenya	2010	SPA	695	80.7	78.5	63.5	85.4	95.1	40.7	27.7	52.3
Namibia	2009	SPA	411	73.7	59.2	64.1	97.6	86.8	92.7	50.7	93.9
Rwanda	2007	SPA	538	80.3	79.8	31.2	93.5	90.8	52.1	2.0	78.1
Senegal	2013	SPA	438	78.1	42.6	62.5	79.6	76.6	1.4	22.9	82.7
Senegal	2014	SPA	452	77.0	46.8	62.0	87.6	89.6	4.5	33.0	93.5
Sierra Leone	2011	SARA	207	89.9	96.6	60.3	81.9	79.6	2.9	19.9	83.5
Sierra Leone	2012	SARA	106	91.5	96.3	86.8	81.7	67.4	3.9	46.1	78.1
Sierra Leone	2013	SARA	455	94.1	88.9	85.0	89.0	93.5	5.4	49.0	90.4
Tanzania	2006	SPA	611	82.2	61.6	50.5	83.8	84.9	42.8	3.7	80.1
Tanzania	2014/15	SPA	1200	85.9	30.9	60.7	88.0	61.1	51.5	32.0	94.9
Togo	2012	SARA	100	92.0	31.4	77.3	80.7	87.8	13.1	10.3	93.5
Uganda	2007	SPA	491	81.3	52.1	32.7	70.1	84.4	26.7	9.3	55.6
Uganda	2012	SARA	95	85.3	64.8	59.6	76.0	83.8	30.9	23.1	86.6
Zimbabwe	2014	SARA	275	95.3	99.2	96.2	91.2	59.9	82.5	71.8	96.2
**Median**			439	84.5	62.0	62.3	82.8	84.1	19.9	23.0	84.9

TT – tetanus toxoid vaccine for pregnant women, IPTp – intermittent preventive treatment of malaria in pregnancy, Syph T&T – syphilis detection and treatment in pregnancy, Hyp – hypertensive disease case management, Fe – iron supplementation

*Source: Service Provision Assessment (SPA) and Service Availability and Readiness Assessment (SARA) surveys.

### Health facility readiness

Among facilities offering ANC services, the percentage with at least one staff member who had recently received in–service training in ANC varied from 30.9% in Tanzania (2014/15) to 99.3% in Zimbabwe (2014). For ANC guidelines, ANC facilities in Zimbabwe (2014) were most likely (96.2%) to report the availability of ANC guidelines compared to less than a quarter (31.2%) of ANC facilities in Rwanda (2007) (**Table 2**). Overall, the percentage of facilities with the necessary equipment, diagnostics, medicines and commodities to deliver IPTp, iron supplementation, and tetanus toxoid vaccination was relatively high. The availability of IPTp drugs exceeded 90% in ANC facilities in DRC (2013), Kenya (2004, 2010), Rwanda (2007), and Sierra Leone (2013). Similarly, more than 90% of ANC facilities in Benin (2013), Namibia (2009), Senegal (2014), Sierra Leone (2014), Tanzania (2014/15), Togo (2012), and Zimbabwe (2014) had iron tablets in stock. By contrast, the availability of equipment, diagnostics, medicines and commodities to deliver hypertensive disease case management and syphilis detection and treatment was relatively low. None of the assessments reported more than 75% of ANC facilities in a country with the capacity to manage hypertensive diseases in pregnant women (median: 23.1%; range: 2.0–71.8%). With the exception of Namibia (2009) and Zimbabwe (2014), less than 90% of ANC facilities had a valid syphilis test and penicillin treatment available on the day of assessment, with a low of 9.3% recorded in Burkina Faso (2012).

### ANC coverage and content

The percentage of women attending at least one ANC visit from a skilled provider (ANC1+) was generally high (median: 93.1%; range: 86.0–97.5%) (**Table 3**). The percentage of women attending at least four ANC visits (ANC4+) ranged from 24.8% in Rwanda (2007/8) to 75.8% in Sierra Leone (2013). Most women first sought care during the second trimester, with the median months of gestation at first ANC visit ranging from 3.7 to 5.9 months. Based on DHS data, more than half of women who attended at least one ANC visit reported blood pressure measurement (median: 93.9%; range: 51.3–99.1%) and receipt of at least one dose of tetanus toxoid vaccine (median: 82.1%; range: 56.7–97.3%). However, the collection of a blood sample (median: 82.7%; range: 28.4–89.3%), the collection of a urine sample (median: 66.9%; range: 11.4–94.1%), the receipt of iron supplementation (median: 79.6%; range: 41.7–94.8%), and the receipt of at least one dose of SP during an ANC visit (median: 41.1%; range: 0.8–73.5%) was much more variable (**Table 3**).

**Table 3:** DHS estimates of coverage of antenatal care (ANC) and ANC interventions among women who reported attending at least one ANC visit*

	Year	ANC1+ (%)	ANC4+ (%)	Early ANC enrollment (%)†	Median months of pregnancy at 1^st^ ANC visit	Blood sample taken (%)	Urine sample taken (%)	Blood pressure measured (%)	Weight measured (%)	Iron tablets or syrup given (%)	Any or complete tetanus protection at birth (%)	Any SP/Fansidar use during ANC visit (%)
Benin	2011/12	86.0	58.3	48.3	3.8	81.3	94.1	97.7	98.3	80.8	78.2	37.3
Burkina Faso	2010	95.5	33.1	40.6	4.3	63.3	84.5	97.2	98.2	93.3	91.0	–
DRC	2013/14	88.5	47.2	16.7	5.4	61.4	52.0	74.1	88.2	58.7	75.0	33.0
Ghana	2003	91.9	68.9	45.8	4.0	85.8	83.7	95.4	94.3	79.4	85.1	0.8
Kenya	2003	87.4	50.9	10.5	5.9	56.4	49.1	82.3	90.9	46.8	85.5	9.9
Kenya	2008/9	91.5	45.7	13.5	5.8	82.7	66.6	84.2	94.0	69.6	88.5	33.6
Namibia	2006/7	94.3	70.2	31.6	4.8	97.3	92.8	97.1	98.0	79.6	56.7	19.6
Rwanda	2007/8	96.1	24.8	23.2	5.3	73.6	17.9	87.0	97.2	41.7	74.1	51.3
Senegal	2012/13	94.2	45.8	54.0	3.7	80.3	86.7	99.1	95.4	93.6	91.2	73.5
Senegal	2014	96.1	47.1	57.8	3.6	85.5	89.3	99.6	95.4	94.8	90.7	71.1
Sierra Leone	2013	97.5	75.8	44.7	4.1	89.3	72.4	93.9	88.7	94.1	97.3	62.1
Tanzania	2004/5	92.3	59.2	13.6	5.5	52.6	39.7	64.0	93.8	61.0	78.8	49.5
Tanzania	2015/16	97.9	49.2	23.1	5.0	87.3	58.6	69.5	93.8	81.9	74.4	68.3
Togo	2013/14	49.5	56.4	27	4.9	87.4	88.4	97.0	N/A	85.8	85.5	58.7
Uganda	2006	93.1	45.9	16.2	5.6	28.4	11.4	51.3	76.5	63.2	76.1	33.5
Uganda	2011	94.9	47.2	20.7	5.2	83.4	22.0	58.0	77.5	75.8	80.9	44.9
Zimbabwe	2015	92.0	74.1	37.4	4.5	98.7	66.9	97.3	94.0	83.9	82.1	13.3
**Median**		93.1	49.2	27.0	4.9	82.7	66.9	93.9	94.0	79.6	82.1	41.1

N/A – no data available

*Reference period: 3 years.

†Early ANC enrollment was defined as first ANC visit before 16 weeks of gestation.

### Likelihood of appropriate care

**Figure 3:**
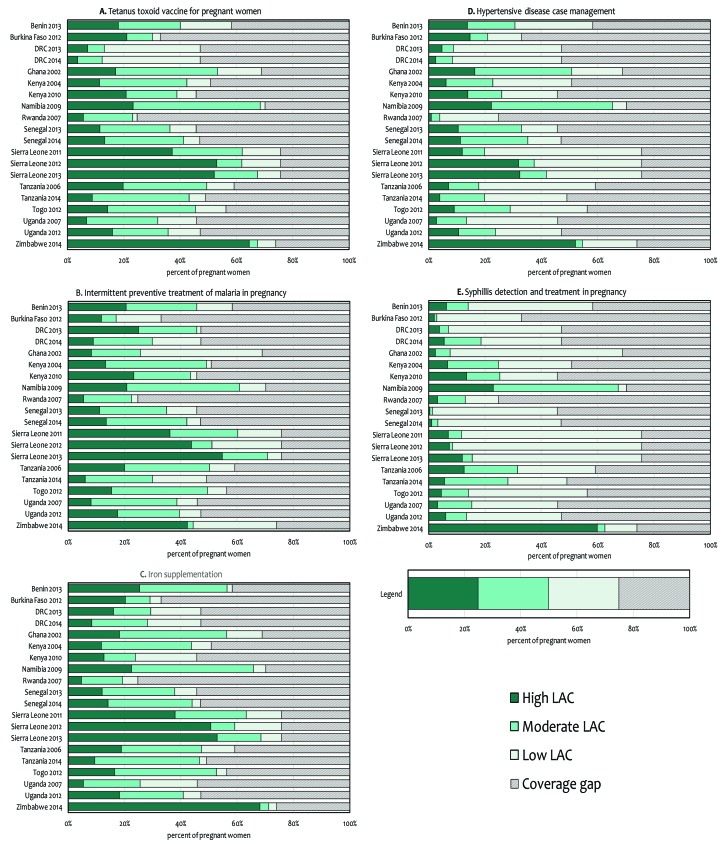
Likelihood of appropriate care (LAC) for antenatal care interventions by country and year.

Based on the results from linking the SPA/SARA and DHS data, we estimated that on average around one in five women with a recent live birth received ANC at a facility with trained staff and appropriate clinical guidelines and IPTp drugs (20.8%), iron supplements (22.5%), or tetanus toxoid vaccine (21.5%) in stock (Table 4). Despite the availability of these three drugs at health facilities, estimates of the likelihood of appropriate care were lower due to underutilization of ANC services, lack of ANC guidelines, and a shortage of trained staff to deliver interventions (**Figure 3**). For example, in Rwanda (2007), more than three quarters of facilities offering ANC services had SP (90.8%), iron supplements (78.1%), or tetanus toxoid vaccine (93.5%) in stock (**Table 2**). However, ANC 4+ coverage was 24.8% (**Table 3**), and only 31.2% of ANC facilities had ANC guidelines (**Table 2**). As a result, very few women had a high likelihood of appropriate care for these three interventions (IPTp: 5.6%, iron supplementation: 4.8%, tetanus toxoid vaccination: 5.8%) (**Figure 3**, **Table 4**).

**Table 4:** Percentage of all pregnant women with a high likelihood of appropriate care for antenatal care (ANC) interventions*

Country	Year	Survey	TT	IPTp	Syph T&T	Hyp	Fe
Benin	2013	SARA	18.1	20.6	6.4	9.2	25.5
Burkina Faso	2012	SARA	21.2	11.9	2.0	3.6	20.3
DRC	2013	SARA	7.2	25.1	3.9	0.9	16.1
DRC	2014	SARA	3.7	8.9	6.7	1.0	8.4
Ghana	2002	SPA	17.3	8.4	2.5	14.5	18.3
Kenya	2004	SPA	11.5	13.3	7.0	0.6	11.9
Kenya	2010	SPA	20.9	23.3	13.5	6.8	12.8
Namibia	2009	SPA	23.4	20.8	23.0	12.2	22.5
Rwanda	2007	SPA	5.8	5.6	3.2	0.1	4.8
Senegal	2013	SPA	11.7	11.2	0.4	3.4	12.1
Senegal	2014	SPA	13.3	13.6	1.1	5.0	14.2
Sierra Leone	2011	SARA	37.3	36.3	7.0	9.0	38.0
Sierra Leone	2012	SARA	53.0	43.8	7.3	29.9	50.7
Sierra Leone	2013	SARA	52.1	54.8	12.0	28.7	52.9
Tanzania	2006	SPA	19.8	20.1	12.7	0.9	18.9
Tanzania	2014/15	SPA	8.8	6.1	5.7	3.2	9.5
Togo	2012	SARA	14.3	15.6	4.5	1.8	16.5
Uganda	2007	SPA	6.8	8.2	3.2	0.9	5.4
Uganda	2012	SARA	16.0	17.6	6.1	4.9	18.2
Zimbabwe	2014	SARA	64.6	42.4	59.9	50.8	68.1
Mean			21.3	20.4	8.4	9.4	22.3

TT – tetanus toxoid vaccine for pregnant women, IPTp – intermittent preventive treatment of malaria in pregnancy, Syph T&T – syphilis detection and treatment in pregnancy, Hyp – hypertensive disease case management, Fe – iron supplementation

*Source: Service Provision Assessment (SPA) and Service Availability and Readiness Assessment (SARA) surveys.

Across countries, the percentage of women attending ANC at facilities ready to perform hypertensive disease case management and syphilis detection and treatment was lower relative to the other interventions, representing substantial missed opportunities for women seeking ANC care. For example, in Sierra Leone (2013) where there was the most widespread utilization of ANC services (ANC4+ coverage: 75.8%) (**Table 3**), only 12.0% of all women with a recent live birth had a high likelihood of appropriate care for syphilis detection and treatment. In countries with multiple health facility assessments (DRC, Kenya, Uganda, Senegal, Sierra Leone and Tanzania), trends over time suggested that there were improvements in the availability of diagnostics for syphilis detection and treatment (**Table 2**), and subsequent improvements in the likelihood of appropriate care for syphilis testing and treatment (**Table 4**). Similar improvements over time were observed for hypertensive disease case management.

**Figure 4:**
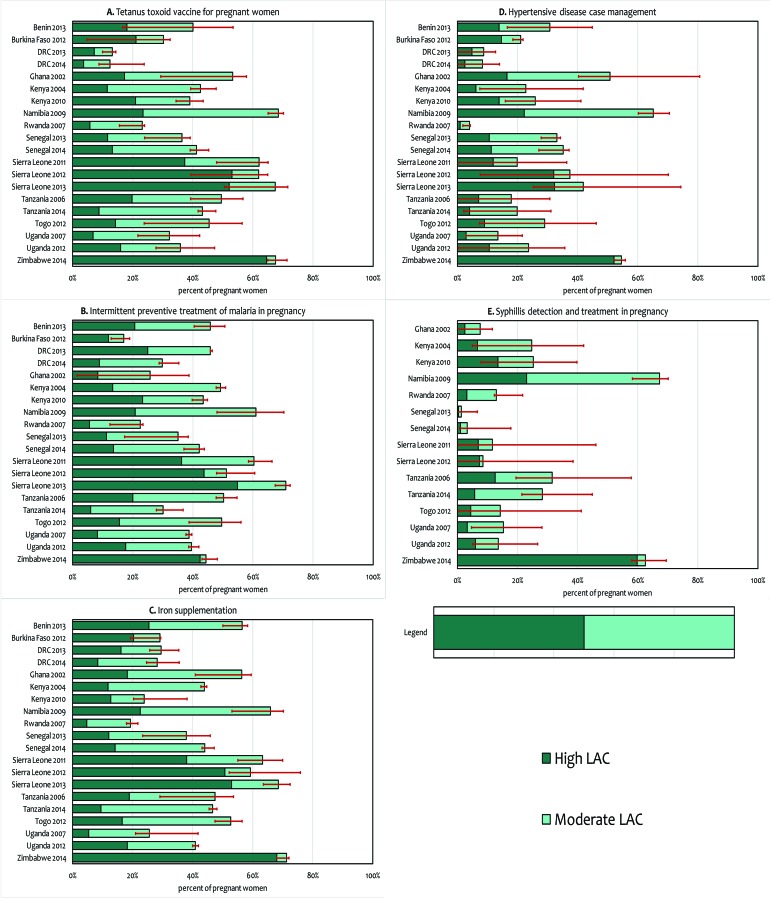
Results of sensitivity analysis to assess the impact of misclassification of stratum on the likelihood of appropriate care (high and moderate).

Sensitivity analyses showed that the overall results were stable and not unduly influenced by potential misclassification of strata (**Figure 4**). However, the sensitivity bounds were wider for syphilis detection and hypertensive disease case management, suggesting a greater heterogeneity in readiness to deliver these interventions by stratum.

**Figure 5:**
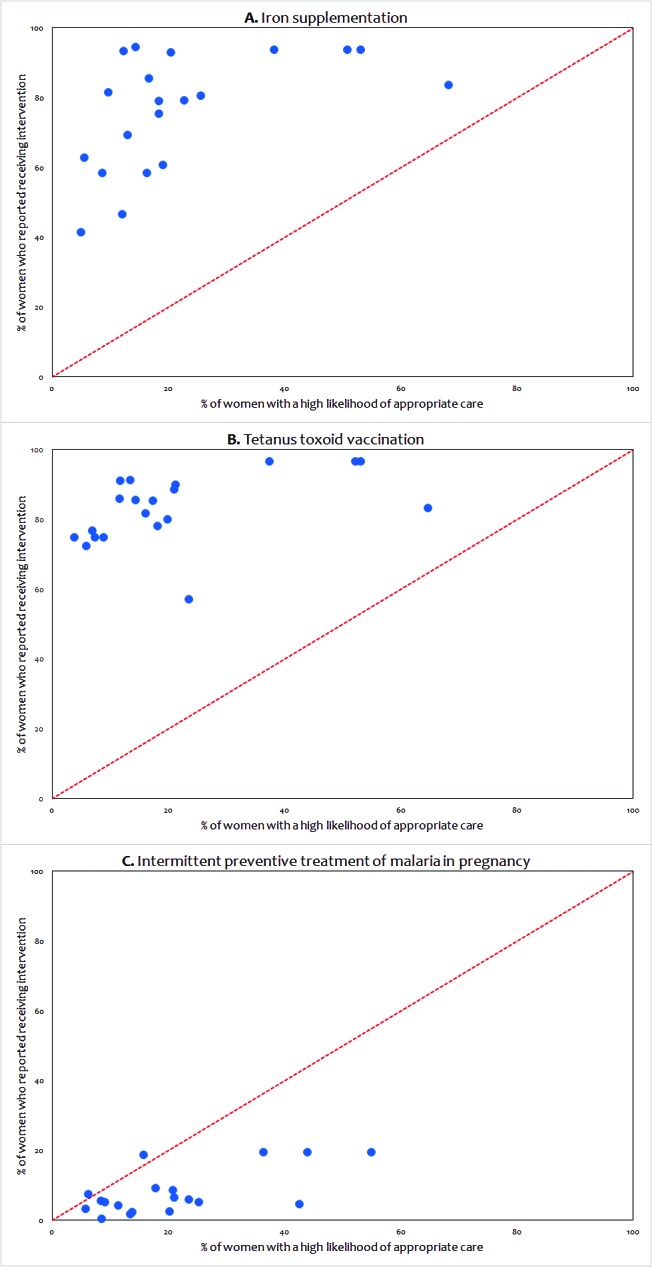
Comparison of estimated coverage (DHS) and estimated high likelihood of appropriate care (LAC) based on the ecological linking for iron supplementation, tetanus toxoid vaccination and intermittent preventive treatment of malaria in pregnancy.

For three interventions, we were able to directly compare estimates of the likelihood of appropriate care based on the linking approach and coverage estimates derived from mother’s recall from the DHS (**Figure 5**). If women sought care at facilities ready to deliver the intervention, and then received it, one would expect “perfect” correlation between these two estimates, assuming women were able to accurately report receipt of the intervention in the household survey. Our estimates of high likelihood of appropriate care correlated relatively well with the DHS coverage estimates for all three interventions (iron supplementation: Pearson correlation 0.52, p value 0.02; tetanus toxoid vaccination: Pearson correlation 0.46, p value 0.04; IPTp: Pearson correlation 0.64, p value 0.003). Our estimates of high likelihood of appropriate care tended to underestimate coverage levels obtained from the DHS for iron supplementation and tetanus toxoid vaccination while our estimates overestimated coverage levels obtained from the DHS for IPTp (**Figure 5**).

## DISCUSSION

This study systematically linked 20 large–scale health facility assessments (SPAs and SARAs) and household surveys (DHS) conducted in sub–Saharan Africa to estimate the percentage of women who attended health facilities which were ‘ready’ to deliver five key ANC interventions. While the immediate goal of our framework was to determine the likelihood of appropriate care for key ANC interventions in several countries in sub–Saharan Africa, our analysis also offers a strategy to identify opportunities for strengthening ANC at the national level. Furthermore, this study underscores the need for a two–pronged approach to improving coverage of ANC interventions: namely, to improve ANC utilization and quality of ANC services.

Household surveys are useful for monitoring trends in ANC coverage based on self–reported utilization patterns. However, ANC coverage does not provide insight about the actual delivery of health services nor does it serve as a reliable measure of coverage of interventions delivered during ANC. Health facility assessments, on the other hand, can provide insight on service availability and readiness at health facilities, but not on population–based coverage. Linking information about service provision or readiness to service utilization data offers great potential in identifying barriers to achieving high population coverage of interventions [27]. For tetanus toxoid vaccine, IPTp, and iron supplementation, our comparison of DHS coverage estimates and linked estimates in this analysis correlated relatively well. While there were biases in the two approaches to measuring coverage of these three interventions, the fairly good concordance suggests that the linking approach may be useful in estimating coverage for those ANC interventions for which DHS provides no coverage estimate.

The percentage of women attending health facilities ready to deliver tetanus toxoid vaccination, IPTp, and iron supplementation was relatively higher than the more complex and multi–step interventions – hypertensive disease case management and syphilis detection and treatment. Hypertension is a major cause of maternal mortality and syphilis is estimated to be associated with 11% of stillbirths [31,32]. Our findings highlight the need to improve commodity–driven logistical barriers for the delivery of ANC services, notably the supply of syphilis tests for screening, injectable penicillin for syphilis treatment, dipsticks for urine protein and magnesium sulphate for pre–eclampsia. Health facility readiness varies by region, health facility type and managing authority, and country specific information is available in the final survey reports [29,30].

There are several limitations when linking health service provision to utilization of health services at an ecological level, as in this analysis. The underlying assumption of our ecological analysis was that women seeking ANC experienced the “average” level of health facility readiness in that stratum, which was not the case. Second, although ideally we would have weighted our readiness estimates by health facility utilization to better reflect the “average” ANC experience, health facility utilization data are not collected in the SPAs or SARAs. Third, the linkage approach relied on women’s self–reported source of ANC care, including the type of health facility and managing authority. To minimize recall bias, our analysis was restricted to women reporting the most recent live birth in the 3 years preceding the survey, but this constraint does not ensure the validity of all responses. Finally, health facility assessments and household surveys may have been conducted at different time points, so that the survey reference period for both sources may not be perfectly matched or temporally aligned. To address this concern, surveys were restricted to occur within two years of each other, with the underlying assumption that ANC utilization patterns were unlikely to change drastically during the two year period. However, given a DHS survey reference period of up to 3 years, it is possible to have a total lag period of up to 5 years between the time a woman had her most recent live birth and the time the health facility assessment was conducted.

Our analysis focused on health facility readiness to provide interventions, which is a minimum requirement for but not a guarantee of the delivery of quality ANC services. While facilities that lack the necessary drugs, equipment, diagnostics or trained staff cannot provide an intervention, ‘ready’ facilities may also fail to provide quality services in part due to lack of provider knowledge, motivation, supervision or increased workloads [27]. Although SPAs provide further information on other dimensions of quality based on observations of ANC consultations and client exit interviews, the SARAs only provide information on service availability and readiness. To allow the use both surveys we focused on health facility readiness. Future research should assess other measures of quality.

Our sample included surveys that were conducted as early as 2002, in order to aggregate a data set for proof of concept, but we acknowledge that changes in the health systems may have occurred in the past decade and recommend caution in drawing inferences based on the cross–country comparisons. Furthermore, health facility readiness can change rapidly over a short time period. Our results may not adequately reflect these shifts or the current situation across all countries in sub–Saharan Africa. As nationally representative surveys are conducted more routinely across more countries, tracking of improvements in the quality of care over time will be possible. Lastly, our analysis did not account for the timing of ANC interventions. Early screening and treatment is a critical consideration for interventions such as syphilis detection and treatment. Our analysis assumed that interventions were appropriately delivered in a timely manner to yield the intended health gains.

Linking ANC readiness and care–seeking data at the population–level was valuable in characterizing the quality of ANC care available to pregnant women and highlighting deficiencies in the provision of ANC services across health facilities in sub–Saharan Africa. The use of standardized survey instruments facilitated the pooling of data from multiple countries and years. We believe that the linking approach that ties utilization to readiness holds great promise to produce estimates of coverage of effective interventions that cannot be or at least are not measured in household surveys.

While there will continue to be work to refine and improve estimates of coverage and service quality with analyses linking household and health facility surveys, two changes could facilitate the easier linking of data from these two sources. First, the inclusion of a question about the source of ANC in the MICS would make allow the inclusion of another standardized source of data on care–seeking patterns. Second, harmonization of the two health facility assessments (SPA and SARA) would further aid linking studies and foster new strategies in order to ultimately improve measurement of coverage in MNCH.
